# Contribution of minced muscle graft progenitor cells to muscle fiber formation after volumetric muscle loss injury in wild‐type and immune deficient mice

**DOI:** 10.14814/phy2.13249

**Published:** 2017-04-11

**Authors:** Benjamin T. Corona, Beth E. P. Henderson, Catherine L. Ward, Sarah M. Greising

**Affiliations:** ^1^ Extremity Trauma and Regenerative Medicine Task AreaUS Army Institute of Surgical ResearchFort Sam HoustonTexas

**Keywords:** Musculoskeletal, orthopedic trauma, regenerative medicine, skeletal muscle injury

## Abstract

Volumetric muscle injury (VML) causes an irrecoverable loss of muscle fibers, persistent strength deficits, and chronic disability. A crucial challenge to VML injury and possible regeneration is the removal of all of the in situ native elements necessary for skeletal muscle regeneration. Our first goal was to establish a reliable VML model in the mouse tibialis anterior (TA) muscle. In adult male wild‐type and nude mice, a non‐repaired ≈20% VML injury to the TA muscle resulted in an ≈59% loss in nerve evoked muscle strength, ≈33% loss in muscle mass, and ≈29% loss of muscle fibers at 28 day post‐injury. Our second goal was to investigate if minced muscle grafts (≈1 mm^3^ tissue fragments) promote recovery of muscle fibers after VML injury and to understand if the graft‐derived progenitor cells directly contribute to fiber regeneration. To assess donor cell contribution, donor muscle tissue was derived from UBC‐GFP mice in a subset of experiments. Minced grafts restored ≈34% of the lost fibers 28 days post‐injury. The number of GFP ^+^ fibers and the estimated number of regenerated fibers were similar, regardless of host mouse strain. The muscle tissue regeneration promoted by minced grafts did not improve TA muscle strength at this time post‐injury. These findings demonstrate the direct contribution of minced muscle graft‐derived myogenic stem/progenitor cells to recovery of muscle fibers after VML injury and signify the utility of autologous myogenic stem cell therapies for this indication.

## Introduction

Insufficient recovery of skeletal muscle strength following orthopedic injury contributes to chronic disability and the estimated ~$400 Billion economic impact of trauma in the United States annually (Bosse et al. 2002; MacKenzie et al. 2005; Corso et al. 2006). The partial traumatic ablation of skeletal muscle, or volumetric muscle loss (VML) injury, occurs frequently in civilian and military trauma (Papakostidis et al. 2011; Corona et al. 2015). This muscle injury is particularly challenging to regenerate due to the removal of all of the in situ native elements necessary for skeletal muscle regeneration (e.g., basal lamina and satellite cells) (Corona et al. 2016). To that end, VML injury currently has no acute regenerative standard of care, and is therefore considered part of the natural sequelae of musculoskeletal trauma (Mase et al. 2010; Garg et al. 2015). In some cases, VML injuries may be treated with free or rotational muscle flaps for the purpose of wound closure, mitigation of infection, or support of concomitant fracture healing, but not restoration of muscle strength. As a result, VML injuries are characterized by a permanent loss of muscle fibers, gross fibrosis, persistent strength deficits, limb dysfunction, and chronic disability (Mase et al. 2010; Corona et al. 2013a,b, 2015; Aurora et al. 2015; Garg et al. 2015; Ward et al. 2015, 2016; Corona and Greising 2016; Rivera and Corona 2016).

Multiple efforts are ongoing to discover an appropriate therapeutic approach to promote de novo muscle tissue regeneration and functional recovery after VML injury. A highly pragmatic approach, due to existing commercial availability and clinical use for other soft tissue indications, is the implantation of an acellular biological scaffold into the VML defect (Sicari et al. 2014). Available data indicate that the capacity of acellular biological scaffolds to regenerate is challenged by restricted satellite cell migration from the remaining host musculature to the defect area (Garg et al. 2014b; Aurora et al. 2015; Corona and Greising 2016), and therefore this approach may require augmentation with myogenic progenitor/stem cells. That being said, autologous satellite cell isolation, expansion, and co‐delivery with an acellular biological scaffold raises multiple biological, practical, and regulatory constraints that limit near‐term clinical application.

Autologous muscle tissue sourcing may serve as a crude but viable method to deliver myogenic cells at the point of care to treat or augment treatment of VML injuries. Autologous minced muscle grafts embody a natural skeletal muscle‐specific biological scaffold, and muscle stem/progenitor (e.g., satellite cells and fibro/adipogenic progenitors) cell delivery vehicle. Minced muscle grafts were investigated extensively in the 1960s–1980s as an injury condition to elucidate foundational concepts of skeletal muscle regeneration. Notably, Carlson demonstrated that whole rat triceps surae muscles minced to ≈1 mm^3^ pieces and orthotopically transplanted maintained the potential to regenerate a whole muscle (albeit of significantly less stature than the uninjured musculature) that regained contractility (Carlson 1968, 1972; Carlson and Gutmann 1972). A host of studies were performed to determine the origin of the myogenic cell, graft versus host. Multiple investigations performed in rats indicated that the muscle progenitor cells were primarily derived from the minced grafts (Ghins et al. 1984, 1985, 1986). In these studies, de novo tissue formation was lost if the minced grafts were devitalized and partially restored with delivery of isolated myogenic progenitors (Ghins et al. 1985, 1986). Recently, the inability of devitalized minced grafts to promote muscle fiber regeneration in VML injured muscle has been reported (Garg et al. 2014b). Direct contribution of minced graft muscle progenitors to de novo muscle fiber regeneration after VML injury has been confirmed using tissue from syngeneic GFP donor rats (Ward et al. 2015). Interestingly, minced grafts have also been shown to support de novo fiber regeneration as a myoconductive element, wherein following complete anterior crural muscle ablation mince graft‐supported regeneration was predominantly attributed to migration of myogenic progenitor cells from the remaining host musculature using donor‐host combinations of mice that express different NADP‐dependent malate dehydrogenase isoforms (Partridge and Sloper 1977). This historical difference in minced graft function parallels current challenges to regenerative medicine solutions for VML, is co‐delivery of myogenic stem cells necessary or can the host myogenic cell migration fully support new muscle tissue formation?

The purpose of this study was two‐fold: First, we sought to establish a reliable VML model in the mouse tibialis anterior (TA) muscle in our laboratory, which would permit in vivo functional testing and the use of transgenic mouse strains in future mechanistic investigations of the pathophysiology of VML injury and therapeutic action. Second, we sought to test the hypothesis that minced muscle grafts promote muscle fiber regeneration after VML injury in mice through direct contribution of graft‐derived muscle progenitor/stem cells.

## Materials and Methods

### Animals

All animal procedures were approved by the Institutional Animal Care and Use Committee and were conducted in compliance with the Animal Welfare Act and in accordance with the principles of the Guide for the Care and Use of Laboratory Animals.

Adult male C57BL/6J (Stock #000664) wild‐type mice were purchased from Jackson Laboratories. Adult male athymic nude mice (*Foxn1* ^*nu*^ *)* were purchased from Envigo (Harlan). Nude mice were included in this study because of their prior use in the VML injury field (Machingal et al. 2011; Corona et al. 2012; Grasman et al. 2015) and to glean initial understanding of the impact of growing role of T lymphocytes in skeletal muscle regeneration on this system (Castiglioni et al. 2015; Schiaffino et al. 2017). Additionally, in a subset of experiments adult male C57BL/6‐Tg(UBC‐GFP)30Scha/J mice (UBC‐GFP; Jackson Laboratories, Stock #004353), which express GFP in all tissues, were used as minced muscle graft tissue donors.

### Experimental design

Two sets of experiments were performed. In the first experiment, both wild‐type and nude mice underwent VML injury and subset of mice subsequently underwent VML injury repair with autologous minced muscle grafts. In the second experiment*,* VML injury in each mouse strain was repaired with minced muscle grafts from UBC‐GFP donor mice, resulting in syngeneic implantation in wild‐type mice. In each experiment, non‐repaired VML injured mice served as negative “injury” controls. All animals were carried out to 28 days post‐injury, at which time in vivo anterior crural muscle functional assessments were performed and then TA and extensor digitorum longus (EDL) muscles were harvested, weighed, and processed for assessments of muscle fiber regeneration. For histological analyses, contralateral TA muscles were used for uninjured controls. For in vivo muscle strength testing, a set of cage control mice (no surgery) was used for uninjured controls. Tissue harvest was conducted following muscle strength testing while mice were deeply anesthetized with (isoflurane 1.5–2.0%) and mice were euthanized with an injection of Fatal Plus (150 mg/kg; intra‐cardiac) while still under anesthesia.

### Surgical creation and minced graft repair of VML injury

The surgical procedure for creating VML injury in the mouse TA muscle was performed as described previously (Pollot and Corona 2016). Briefly, with aseptic technique a lateral incision along the lower limb was created to gain access to the TA muscle. A VML defect was created in the middle third of the TA muscle in the left leg using a 2 mm biopsy punch. Defect weights for each experimental group were recorded. As per experimental condition, TA muscles were repaired with minced muscle grafts, which were created from either the piece of TA muscle excised to create the VML defect (autologous) or donor TA muscle from UBC‐GFP mice by mincing the tissue using Vanna scissors into 1 mm^3^ fragments. The minced tissue was then placed into the fresh wound bed within 10 min of preparation (Ward et al. 2016). The muscle graft repair was 100% of the removed tissue. Mice were administered buprenorphine‐HCl, 1.0 mg/kg subcutaneously approximately 30 min before surgery and at 12 and 24 h post‐surgery for post‐operative pain. Following surgery all mice recovered promptly and displayed only slight limitations in mobility. No unexpected deaths or adverse outcomes were noted in any experimental groups.

### Quantitative histology

TA muscles were embedded in OCT and frozen in liquid nitrogen cooled melting isopentane as described previously (Corona et al. 2008; Corona et al. 2010). TA muscles were cut into 8 *μ*m cross‐sections and stained with Hematoxylin and Eosin using standard staining procedures. Cross‐sections of TA muscles with UBC‐GFP donor tissue were stained with a monoclonal GFP antibody (1:100, Invitrogen, G10362) followed by an Alexa Fluor 488 conjugated secondary antibody (1:500, Invitrogen, A‐11034). Cross sections were viewed by brightfield microscopy and with mercury illumination (Olympus IX71) and images of muscle tissue were acquired and analyzed using cellSens Standard software (Ver 1.4.1, Olympus). Stitching on individual image frames to the whole muscle composite image was completed using Adobe Photoshop (Adobe Systems Inc.). Composite images were used to manually analyze the total muscle fiber number and GFP^+^ fiber number using ImageJ software. Pilot testing in an analogous rat TA muscle VML model demonstrated a stable magnitude of whole muscle fiber reduction from 3 to 21 days post‐injury (*data not shown*). Therefore, estimated “regenerated” fiber number was calculated by subtracting the average fiber number of non‐repaired VML injured muscles from the total fiber number of each minced graft repaired muscle. Additionally, because autologous minced muscle grafts promote muscle fiber formation by initially undergoing degeneration of the mature muscle fragments (Carlson 1972; Carlson and Gutmann 1972; Snow 1977a,b), all GFP^+^ fibers were considered a derivative of donor tissue muscle progenitor/stem cells.

### In vivo muscle strength

Anterior crural muscle in vivo isometric torque was measured in anesthetized mice (isoflurane 1.5–2.0%) in the left leg as previously described (Corona et al. 2010). Core body temperature was monitored and maintained at 36–37°C. Using tape, the foot was strapped to a footplate attached to a dual‐mode muscle lever system (model 300c; Aurora Scientific). The knee was secured on either side using a custom‐made mounting system, and the knee and ankle were positioned at right angles. Platinum percutaneous needle electrodes were place across the common peroneal nerve and connected to a Grass S88 stimulator via a stimulus isolation unit (Grass Instruments; SIU5). Optimal voltage (2–5 V) was then set with a series of tetanic contractions (5–10 contractions; 300 Hz, 0.1‐msec pulse width, 200‐msec train). Maximal isometric torque of the anterior crural muscle unit [TA, EDL, and extensor hallucis longus (EHL) muscles] was derived from this testing. Then, a skin incision was made at the anterolateral aspect of the ankle and the distal EDL muscle tendon, and the EHL muscle was isolated and severed. The TA muscle and tendon, as well as the retinaculum, were undisturbed. Four to five tetanic contractions were performed to allow for torque stabilization and to ensure voltage optimization. Maximal TA muscle isometric torque was then determined using identical stimulation parameters listed above. Previous pilot testing indicated that the contribution of the EDL muscle to net torque is negligible under these conditions (Corona et al. 2013a). Isometric torque is expressed as Nmm per kg body weight to determine absolute functional capacity or Nmm per g muscle wet weight (TA and/or EDL muscles) to assess functional quality.

### Statistics

Dependent variables were analyzed separately using a variety of ANOVAs. Upon observing significance, Fisher's post hoc means comparisons testing was performed. Alpha was set at 0.05. Group responses are presented as box (25–75 percentile with median line) and whisker (minimum and maximum response) plots. Values presented within the text are either approximated percentages or means ± SEM. Statistical testing and graphing was performed using Prism 6 for Mac OSX (Graphpad Inc.).

## Results

### Body and muscle weights

The nude mice weighed significantly more than the wild‐type mice at surgery and sacrifice, although for each strain there were no differences in body weights among cage control, no repair, and minced graft repair groups (Table**** 1). Despite using a uniform 2 mm biopsy punch to create the VML defect, the amount of tissue removed varied by 1.5–1.8 mg among groups. There were no significant differences between strains or among experimental groups in uninjured (contralateral, right leg) TA muscle weight. Moreover, there were no significant differences between strains or between non‐repaired and minced graft‐repaired VML injury groups in injured TA muscle weight. That being said, both wild‐type and nude mice presented a 30–33% reduction in injured TA muscle wet weight compared to uninjured (contralateral, right leg) TA muscles regardless of repair. EDL muscle wet weights were not different between strains or among groups for uninjured (contralateral, right leg) or injured muscles (left leg). The ratio of injured to uninjured EDL muscle weights were also not statistically different among conditions, although both minced graft repair groups presented a tendency to increase (Table 1).

**Table 1:** Mouse body and muscle weights

	Wild Type						Nude					
	Cage control		No repair		Minced graft		Cage control		No repair		Minced graft	
	Right	Left	Right	Injured	Right	Injured	Right	Left	Right	Injured	Right	Injured
Body Weight (g)												
Surgery	–		27.9 ± 0.3		27.7 ± 0.8		–		35.1 ± 0.9^a^		36.0 ± 0.7^a^	
Sacrifice	29.2 ± 0.4		27.3 ± 0.2		28.5 ± 0.6		37.1 ± 1.4^a^		36.2 ± 1.0^a^		36.3 ± 1.0^a^	
TA Muscle Defect (mg)	–		9.9 ± 0.5		8.1 ± 0.3		–		8.6 ± 0.5^a^		10.1 ± 0.5^a^^,^ b	
TA Muscle Weight (mg)	55.9 ± 1.3	57.2 ± 1.2	57.0 ± 1.3	38.9 ± 2.6^c^^,^ d	55.8 ± 1.5	37.4 ± 3.0^c^^,^ d	57.7 ± 2.5	59.0 ± 2.7	55.8 ± 2.8	39.5 ± 5.6^c^^,^ d	59.4 ± 2.3	41.5 ± 4.6^c^^,^ d
Left(Injured):Right	1.02 ± 0.01		0.68 ± 0.04^c^		0.67 ± 0.05^c^		1.02 ± 0.03		0.70 ± 0.08^c^		0.70 ± 0.07^c^	
EDL Muscle Weight (mg)	12.2 ± 0.4	12.4 ± 0.4	12.5 ± 0.6	12.7 ± 0.5	12.7 ± 0.4	13.6 ± 0.6	12.8 ± 0.6	13.1 ± 0.4	12.6 ± 0.4	12.1 ± 0.8	12.0 ± 0.6	13.6 ± 0.8
Left(Injured):Right	1.01 ± 0.02		1.03 ± 0.05		1.07 ± 0.03		1.03 ± 0.03		0.98 ± 0.08		1.15 ± 0.09	

Values are means ± SEM.

a Different from corresponding wild type.

b Different from respective strain no repair.

c Different from strain cage control.

d Different from group right leg; *P* < 0.05.

### TA muscle fiber number

**Figure 1:**
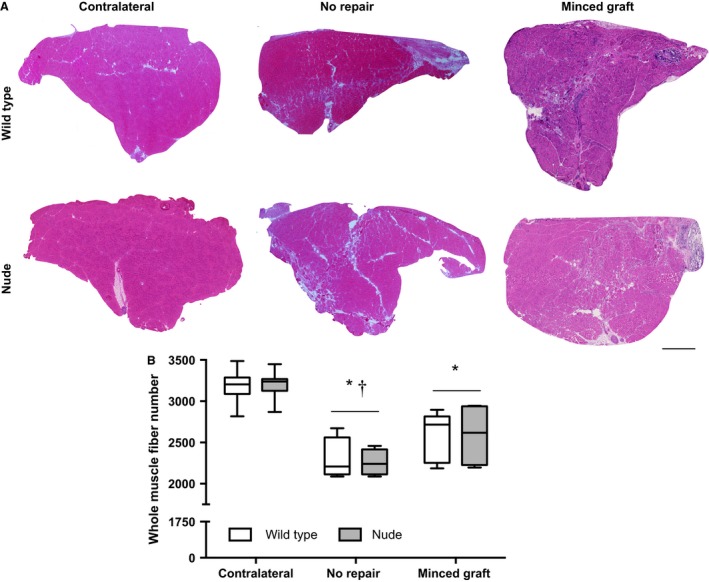
Autologous minced muscle grafts partially restore muscle fiber number after repair of VML injury. TA muscles were harvested at 28 days post‐injury. (A) Representative whole TA muscle cross‐sections stained with Hematoxylin and Eosin are presented per mouse strain and experimental group. Scale bar = 400 *μ*m. (B) Whole TA muscle fiber number plotted per mouse strain and experimental group (Sample Sizes [Wild Type, Nude]: Contralateral (9, 7), No Repair (4, 4), and Minced Graft (5, 4). Two‐way ANOVA demonstrated only a main effect of experimental group (*P *< 0.001) and not a main effect of mouse strain (*P* = 0.977) or an interaction (*P* = 0.965). *, <Contralateral; ^†^<Minced Graft; *P* < 0.05.

The total number of muscle fibers within cross‐sections derived from the midbelly (uninjured) or defect region was counted in uninjured (contralateral) and injured non‐repaired and autologous minced graft‐repaired TA muscles harvested 28 days post‐injury from wild‐type and nude mice. No significant differences between strains were observed. VML injury left unrepaired resulted a ≈29% decrease in the total number of TA muscle fibers (Fig. 1). Autologous minced graft repair of the VML injury promoted a ≈14% increase compared to non‐repaired muscles, leaving a residual ≈19% loss of muscle fibers compared to uninjured contralateral muscles.

**Figure 2:**
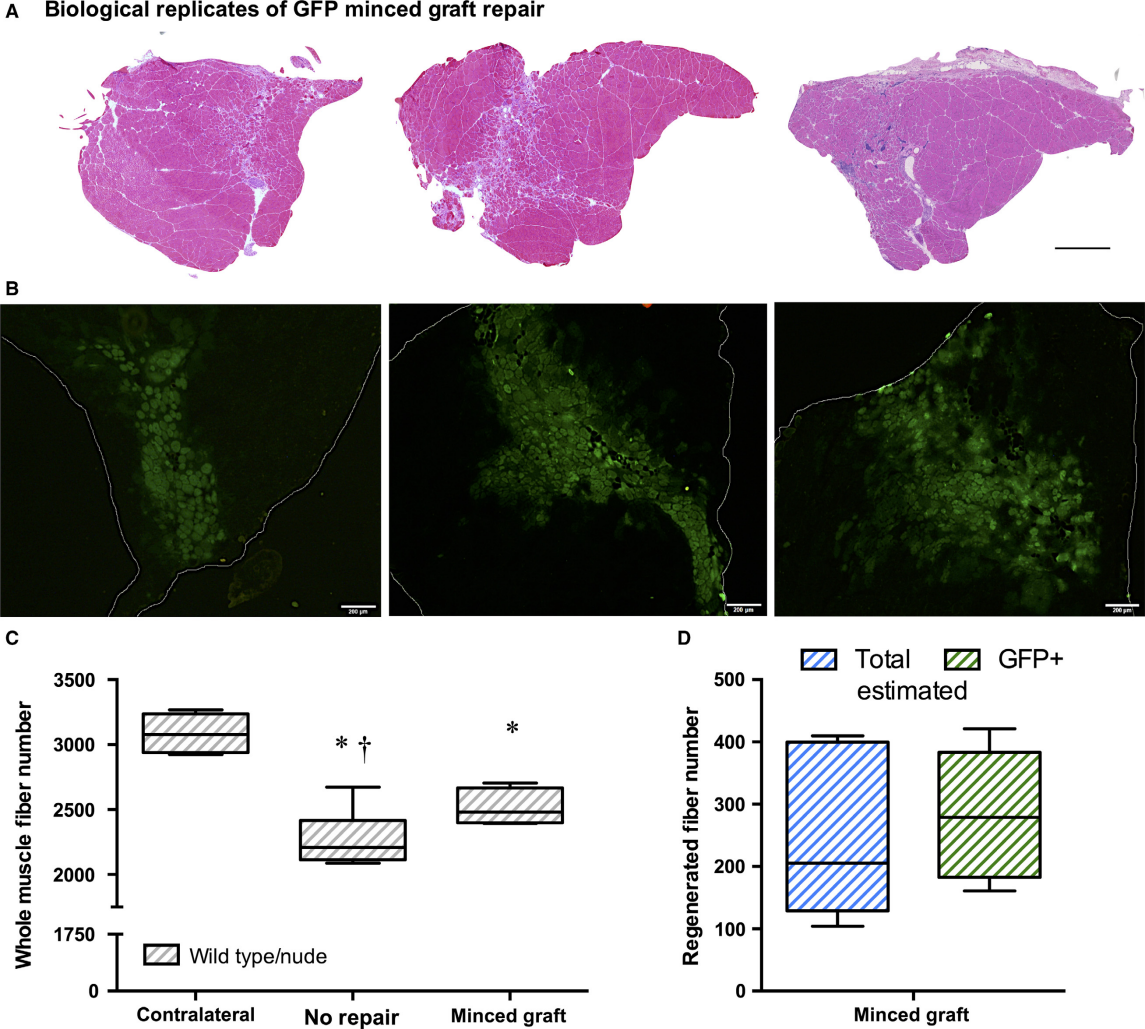
Minced muscle graft‐derived muscle progenitor cells directly contribute to muscle fiber regeneration after repair of VML injury. TA muscles were harvested at 28 days post‐injury. (A) Biological replicates of whole TA muscle cross‐sections stained with Hematoxylin and Eosin are presented from wild‐type mice with UBC‐GFP minced graft repair. Scale bar = 400 *μ*m. (B) Corresponding images of GFP expression in each replicate shown above. White lines define the boundaries of the cross‐section. Scale bar = 200 *μ*m. (C) Whole TA muscle fiber number plotted per experimental group with wild type and nude observations collapsed based on a separate statistical analysis (Sample Sizes [Wild Type, Nude, Group Total]: Contralateral (3, 3, 6), No Repair (4, 4, 8), and Minced Graft (3, 3, 6). The No Repair group data is represented in this and Fiure** 1 for comparison. One‐way ANOVA demonstrated an experimental group effect (*P* < 0.001). *, <Contralateral; †, <Minced Graft; *P* < 0.05. (D) Total estimated (see methods) number of regenerated muscle fibers and GFP ^+^ muscle fibers. No statistical difference was observed (*P* = 0.232).

To assess the contribution of minced graft donor cell contribution to muscle fiber regeneration after VML injury, a second experiment was performed in which minced grafts were derived from UBC‐GFP mice. When assessing the total number of muscle fibers in contralateral, injured non‐repaired, and injured minced graft‐repaired groups from wild‐type and nude mice, the results were similar to those observed with autologous repair. There were no differences between strains. Non‐repaired muscles presented a ≈26% decrease in fiber number and UBC‐GFP minced graft repair promoted a ≈11% increase compared to non‐repaired muscles, leaving a ≈18% residual loss of fibers compared to contralateral controls (Fig. 2). In these samples, the estimated number of fibers regenerated by minced graft repair was 243 ± 55 fibers. The number of GFP^+^ fibers counted in each UBC‐GFP repaired TA muscle was 284 ± 46 fibers, indicating that the muscle progenitor cells in donor minced muscle grafts were the primary contributor to muscle fiber regeneration in VML injured muscle (Fig. 2).

### In vivo isometric torque

**Figure 3:**
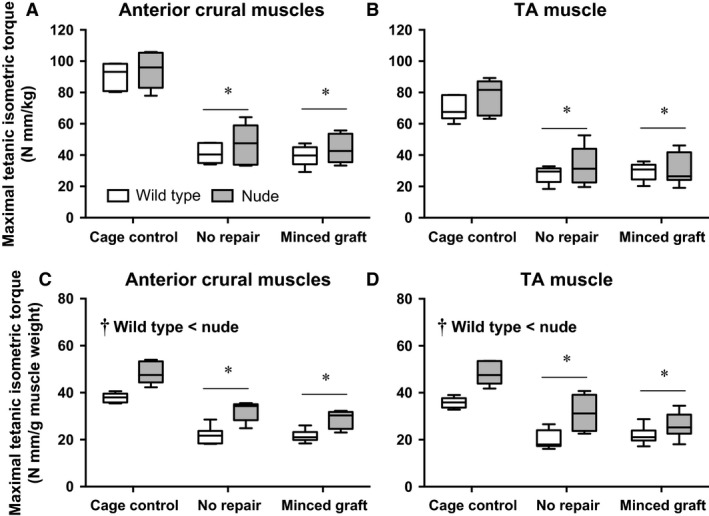
In vivo TA muscle isometric strength is significantly reduced by Volumetric muscle injury and not improved by minced graft repair. Anterior crural muscles and isolated TA muscle maximal isometric torque was measured in wild‐type and nude mice 28 days post‐injury (Sample Sizes [Wild Type, Nude]: Cage Control (5, 5), No Repair (7, 6), and Minced Graft (6, 7). (A & B) Functional capacity was determined by normalizing torque to body weight (Nmm/kg body weight). Two‐way ANOVA demonstrated only a main effect experimental group (*P* <0.001) and not a main effect of mouse strain (*P* = 0.123 & 0.140) or an interaction (*P* = 0.979 & 0.737). *, < Cage Control; *P* < 0.05. C & D) Functional quality was determined by normalizing torque to muscle wet weight (Nmm/g muscle wet weight). Two‐way ANOVA demonstrated a main effect of experimental group (*P* ≤ 0.001), a main effect of strain (†,*P* < 0.001), but not an interaction (*P* = 0.461 & 0.114). *, < Cage Control; *P* < 0.05.

Anterior crural and isolated TA muscle absolute functional capacity was assessed at 28 days post‐injury in wild‐type and nude mice (Fig. 3A and B). There were no significant differences in maximal isometric torque between strains. Non‐repaired injured muscles presented a ≈55% and ≈59% reduction of anterior crural muscle and isolated TA muscle strength, respectively. The functional capacity deficits were not ameliorated by minced graft repair. Additionally, the functional quality of the musculature was assessed by normalizing torque to muscle wet weight (Fig. 3C and D). Collapsed across all conditions, wild‐type mice were significantly weaker than nude mice. Additionally, all non‐repaired injured muscles presented a ≈39% and ≈41% reduction of anterior crural muscle and isolated TA muscle torque per gram of muscle, respectively, regardless of mouse strain or minced graft repair.

## Discussion

The most salient finding of this study was that muscle stem/progenitor cells delivered via minced muscle grafts to a VML defect in the mouse TA muscle are the primary contributor to the observed increase in muscle fiber number. That is, herein we observed that VML injury reduced the number of muscle fibers in the TA muscle by ≈29% and that minced graft repair restored ≈34% of the lost fibers. Using UBC‐GFP donor muscle tissue, the estimated number of fibers regenerated and the number of GFP^+^ fibers in TA muscle cross‐sections were similar. These results indicate a direct contribution of donor derived myogenic stem/progenitor cells, but do not rule out contributions of host myogenic cell migration. Given the dearth of muscle fiber regeneration typically observed in whole and partial (i.e., VML) muscle ablation models when devitalized or acellular biological scaffolds are implanted (Ghins et al. 1984, 1985, 1986; Schultz et al. 1986; Corona et al. 2013b; Garg et al. 2014b; Aurora et al. 2015), the current findings suggest that co‐delivery of a myogenic stem cell source is necessary to recover muscle fiber number after VML injury.

An objective of this study was to develop a mouse VML injury model in our laboratory to facilitate future mechanistic investigation of the pathophysiology of VML injury. By definition, we achieved this goal as the TA muscle defect resulted in a persistent loss of strength (≈59%), wet weight (≈33%), and fiber number (≈29%) at 28 day post‐injury if left unrepaired. The strength deficit following VML injury was significantly greater in magnitude than the loss of wet weight and fiber number, which is observed consistently across multiple VML models (for review see Corona et al. 2016), suggesting that disruption to architectural and potentially neural structures also contributes to the functional deficits. Additionally, the functional quality of the musculature, assessed as torque per muscle wet weight, was significantly reduced (≈40%), which in addition to the aforementioned perturbations to the muscle also reflects accrual of greater fibrotic tissue content (Garg et al. 2014a, 2015). This model presents many of the known pathophysiological features of VML and will likely prove informative in identifying novel therapeutic targets for VML injuries.

Previous reports have indicated that minced grafts may promote de novo muscle fiber regeneration by principally providing a myogenic cell source or by serving as a myoconductive scaffold. Using models where whole muscles were ablated, minced, potentially manipulated (e.g., freeze‐thaw devitalization), and then orthotopically transplanted, the contributions of host and donor muscle progenitor/stem cells were evaluated. Specifically, investigations in which minced muscle grafts were manipulated to vary the level of muscle stem cell delivery indicated a corresponding magnitude of muscle fiber regeneration (Ghins et al. 1984, 1985, 1986). Notably, when only devitalized grafts were implanted (i.e., no muscle progenitor/stem cells), no muscle fiber regeneration was observed, unless freshly isolated or cultured muscle progenitor cells were co‐delivered. In contrast, investigations using donor‐host tissue combinations from mice that expressed different isoforms of NADP‐MDH, which permitted host cell muscle contribution, demonstrated that the majority of muscle regeneration was due to host cell contribution (Partridge and Sloper 1977). The latter findings are interesting in the context of ascribing a host source of muscle stem cells. In the whole muscle ablation model used, the myogenic stem cell would have to be derived from either the vasculature, which rules out satellite cell inclusion (Lepper et al. 2011), or the surrounding musculature, which is unlikely due to restriction of satellite cell migration across the epimysium (Schultz et al. 1986). Regardless, in VML injuries it is highly plausible that satellite cells may migrate from the remaining musculature to a myoconductive scaffold. Yet, even in these optimal conditions, host cell orchestrated muscle regeneration is extremely low (Corona and Greising 2016). The current study and others (Corona et al. 2013a; Garg et al. 2014b; Ward et al. 2015; Kasukonis et al. 2016) have demonstrated muscle fiber regeneration following minced graft implantation in a VML defect, and herein we have definitively demonstrated a significant contribution of donor muscle progenitor/stem cell in this mouse model. Collectively, these findings support the biological requirement of myogenic stem/progenitor cell co‐delivery with a myoconductive scaffold to achieve appreciable muscle fiber regeneration after VML injury, for example, approximately one‐third of the muscle fibers lost after injury (Figs. 1, 2).

In previous studies, the repair of TA muscle VML injury using minced grafts has promoted significant functional improvements at 2–4 months post‐injury (Corona et al. 2013a; Garg et al. 2014b; Kasukonis et al. 2016). However, minced graft repair of the quadriceps did not improve strength earlier post‐injury (~1 month) despite some histological indication of muscle fiber regeneration (Li et al. 2014). Notably, in this model removal of approximately 10% of the quadriceps resulted in an ≈70% functional deficit, which the authors contributed to co‐morbid peripheral nerve injury. Our observation of no functional improvement in this mouse TA VML injury model may therefore be due to inadequate integration with remaining host musculature at 28 days post‐injury. The VML injury created in this study is likely to incorporate concomitant intramuscular nerve damage given its location and full thickness and therefore may face similar challenges to recover strength as observed after muscle laceration (Lim et al. 2006; Pereira et al. 2006) and quadriceps VML injury (Li et al. 2014). Alternatively, it is possible that the approximate 300 regenerated muscle fibers did not produce enough force, potentially due to undeveloped excitation‐contraction coupling machinery, or were not arranged in a manner to meaningfully contribute to net muscle torque.

Unlike dystrophic muscle conditions, autologous myogenic cell therapies for trauma patients are not necessarily encumbered by known pathologic gene mutations. However, given the emerging role of the adaptive immune response to severe muscle trauma (Hurtgen et al. 2016; Sadtler et al. 2016) and muscle regeneration, the regenerative potential of minced muscle grafts was investigated in nude mice depleted of T‐lymphocytes and T Natural Killer cells. For instance, CD8^+^ T cells may also have a positive impact on satellite cell proliferation, which may be related to CD8^+^ T cell‐mediated CCL2 (MCP‐1) secretion and infiltration of Gr1^high^ macrophages (Zhang et al. 2014). Also, regulatory T cells (Tregs) may actively suppress exacerbated pro‐inflammatory responses (Schiaffino et al. 2017), and may also promote regeneration of skeletal muscle by acting on satellite cells (Burzyn et al. 2013; Matta et al. 2014; Arpaia et al. 2015). That being said, regenerative indices such as muscle fiber number and isometric torque were not different between wild‐type and nude mouse strains for any experimental condition in this study. The findings suggest that the adaptive immune response does not impact muscle fiber regeneration using autologous tissue and may not play a significant role in prolonged strength deficits after VML injury, though targeted studies are certainly needed to confirm these initial findings and to interrogate the utility of immunomodulatory therapies to enhance the contribution of innate and adaptive immune responses to muscle tissue regeneration.

Clinical repair of VML injuries is a complex endeavor that will require a variety of regenerative therapies and approaches. In considering the goal of regenerating a large volume of muscle tissue, all approaches will likely be challenged by vascular and neural host integration. This study specifically describes an autologous muscle tissue sourced approach that directly contributes muscle progenitor/stem cells to the recovery of muscle fiber regeneration after VML injury. While this approach has various potential biological and practical strengths, autologous minced muscle grafts are currently limited to the repair of small VML defects due to availability of and morbidity with sourcing autologous muscle tissue. However, the strengths and weaknesses of autologous minced muscle grafts and acellular biological scaffolds may complement each other, wherein biological scaffolds may offer volume expansion to minced grafts and the minced grafts provide a in situ source of myogenic progenitors and stem cells (Ward et al. 2015; Kasukonis et al. 2016).

## Conclusions

Induced de novo tissue regeneration following traumatic or surgical loss of skeletal muscle remains a significant challenge. However, the field is advancing, both in understanding of the underlying cellular and molecular mechanisms manifesting pathobiology and physiology of traumatized muscle. With these advancements innovation in therapeutic approach and design is certainly forthcoming. One of the major questions in the field is whether delivery of a myogenic stem cell source is necessary to promote recovery of muscle fibers after VML injury, or whether host myogenic stem cells can repopulate and instigate regeneration within a myoconductive scaffold. This study demonstrates that donor muscle progenitor/stem cells directly contribute to a significant recovery of muscle fibers, and therefore encourages continued discovery of muscle resident myogenic stem and progenitor cell therapies to induce de novo muscle tissue regeneration after VML injury.

## Conflict of Interest

The opinions or assertions contained herein are the private views of the authors and are not to be construed as official or as reflecting the views of the Department of the Army or the Department of Defense. The authors have no perceived or potential conflicts of interest to disclose, financial or otherwise.
